# Response to “Assessing the role of Piscine orthoreovirus in disease and the associated risk for wild Pacific salmon”

**DOI:** 10.1186/s12915-023-01600-7

**Published:** 2023-05-19

**Authors:** Mark P. Polinski, Yangfan Zhang, Phillip R. Morrison, Gary D. Marty, Colin J. Brauner, Anthony P. Farrell, Kyle A. Garver

**Affiliations:** 1 U.S. Department of Agriculture – Agriculture Research Service, National Coldwater Marine Aquaculture Center, Orono, ME 04469 USA; 2 Department of Organismic and Evolutionary Biology, Harvard University, Cambridge, MA 02138 USA; 3 Biology Department and Department of Resource Management and Protection, Vancouver Island University, Nanaimo, BC V9R5S5 Canada; 4 Animal Health Centre, Ministry of Agriculture and Food, Abbotsford, BC V3G2M3 Canada; 5 Department of Zoology, University of British Columbia, Vancouver, BC V6T1Z4 Canada; 6 Faculty of Land and Food Systems, University of British Columbia, Vancouver, BC, V6T1z4 Canada; 7 Pacific Biological Station, Fisheries and Oceans Canada, Nanaimo, BC V9T6N7 Canada

**Keywords:** Piscine orthoreovirus, Salmon, Respiratory performance

We thank Mordecai et al. [1] for their interest in our work [2] and for their acknowledgment of its comprehensive nature. Indeed, this is the first examination of so many respiratory parameters to test functional impacts of a fish virus. All the same, the large number of parameters measured in our study enables diversity for conducting statistical analyses and we appreciate alternative statistical perspectives.

We feel it important, however, that offered perspectives have fundamental groundings in functional importance and the inherent biological variability associated with measuring respiratory parameters. Equally important is a recognition that the respiratory and oxygen transport indices in our dataset are measures of an integrated and complex biological system for which redundancies are expected as is the case for most physiological systems. Thus, statistical analyses and conclusions/perspectives focusing on variations of isolated parameters — for which Mordecai et al. suggest we have overlooked regarding PRV — ignores the holistic and allostatic nature of the vertebrate respiratory system. More seriously, it runs the risk of defining “harm” or “disease” in association with changes that may not ultimately affect organism functioning.

A good example of isolated-metric variation was for EPOC (the oxygen used during recovery from exhaustion), which Mordecai et al. singled out because EPOC was statistically reduced at a single time point (1 Wpc) following PRV injection. Missing from this overly focused analysis is a crucial fact: control EPOC has a very large 95%CI (over a 2-fold range; Fig. S1 [2]) because EPOC is innately variable. Spontaneous activity sometimes manifests during the recovery process of salmon irrespective of experimental manipulations. It is therefore important to also consider maximum aerobic capabilities, which were virtually identical between treatments in this instance (ṀO2_max_ was numerically highest at 1 Wpc in PRV fish, a potential benefit!). Thus, aerobic capacities during exhaustive exercise were uncompromised by PRV while the oxygen used during recovery was putatively reduced. A true suppression of EPOC would most likely require a depletion of muscle glycogen stores, which we did not measure. Without this information, we could speculate, but did not, that PRV fish were simply a calmer cohort than the control fish during recovery, thereby reducing variance towards high EPOC values. We therefore disagree with Mordecai et al. in speculating functional harm from this specific, unsustained statistical difference to its time-matched control given our larger data set.

Mordecai et al. also singled out PRV exposure as putatively lowering SMR across treatments and time. Unlike for IHNV, however, SMR was not correlated with PRV load as their Fig. 1 shows [1]. Also, PRV did not affect O_2crit_, a metric which is partly dependent on SMR. Furthermore, if as hypothesized by Mordecai et al. that this change had functional significance, it should be reflected at another level of biological organization — specifically, host transcriptomic signaling and/or altered infection dynamics during IHNV superinfection for which reduced SMR appears to be correlated with resistance. This has not been observed in independent challenge investigations. PRV has not affected metabolic transcriptomic signaling [3], nor has PRV altered IHNV load or morbidity/mortality outcomes during superinfection for which reduced SMR would be expected to provide a functional benefit [2, 4]. Thus, we conclude that it is unlikely that PRV functionally impacted basic metabolic requirements of host salmon as measured by SMR.

Our study did indicate that PRV loads were negatively correlated with Hct and Hb for which we hypothesized host-directed removal of highly infected red blood cells or increased red blood cell fragility as possible explanations. Nevertheless, a transient statistical drop in Hct at 4 Wpc was not reflected in Hb concentration which is a functionally more important measure of maximum oxygen transporting capacity than Hct (although Hct is the easier of the two to measure) — see Fig S2. Biologically speaking, Hct and Hb of teleost fish are not only plastic (to optimize oxygen delivery while minimizing cardiac energy demands [5]), they are highly labile to handling perturbations. Therefore, these statistical changes must be placed in a biological context by juxtaposing the Hct values of PRV-infected tank fish in this study (mean 44% ± 5% SD; minimum 35%) relative to a previously proposed lower threshold for functional harm to prolonged swimming and ṀO2_max_ in a salmonid. This proposed threshold is when Hct is < 22% [6], an appreciable reduction afforded presumably through redundancies in the oxygen transport system. This provides evidence that energetic homeostasis was functionally maintained during PRV infection if Hct averaged 44%. Indeed, our respiratory data confirmed this. ṀO2_max_, O_2crit_, and ILOS were not compromised in PRV-injected fish despite a statistical decrease in Hct. We stand by our original statement that any putative harm from reductions in Hct and Hb coinciding is at most a small and temporary effect of PRV infection. An extended EPOC_dur_, an equally variable parameter as EPOC, might have been a measurable aerobic cost. However, the timeframe to manifest harm relating to exhaustive chase recovery is short. Specifically, high viral loads sufficient to incur a putative EPOC_dur_ extension last for a few weeks at most, the added putative time required to recover from exhaustion during peak infection is a matter of hours (mean ~ 4 h increase; maximum ~ 11 h increase), and a repeated bout of maximal exhaustive exercise would likely be needed during this EPOC period to produce a measurable aerobic cost [7].

Our stated hypotheses were clearly focused on the general energetic consequences of the teleost antiviral defense, and so our statistical and power analyses centered on correlative agreements in concert with analysis of variance across treatments and time using GraphPad Prism — a reputable commercial statistical and graphing program. Given the complexity and interconnectedness of our respiratory metrics, our comprehensive evaluation was further framed in a context of biological relevance for which we considered inherent variability in control populations and couched our conclusions with a current and well-recognized understanding of salmonid respiratory plasticity.

We agree with Mordecai et al. that secondary Dunnett post hoc tests presented in our supplemental material have limited statistical power. Indeed, probability estimates were intentionally presented in this context without false-discovery rate adjustment to demonstrate relaxed type-1 and stringent type-2 error controls as a partial means of mitigating the increased potential for type-2 error associated with low power. Moreover, these analyses provided a visually appealing way to present time- and treatment-specific variability, as well as a cautious means for specifying temporal and pathway-specific resolution identified by ANOVA. Probability estimates (and particularly any arbitrary cutoff values associated with probability estimates) generated during these analyses were not, however, intended to define or speculate on biological relevance and we did not use them for that purpose. We would caution others not to do so as well, as discussed above, without fully providing context to functional importance, inherent biological variability, and technical challenges associated with specific measurements.

We certainly agree with Mordecai et al. that increasing statistical power in defining time-specific treatment variation of post hoc Dunnett tests would aid in identifying minor treatment effects on respiratory parameters including those putatively altered by PRV — i.e., increasing certainty of small virus-specific variations at the sensitivity of post hoc testing. However, as acknowledged by Mordecai et al., the potential benefits must be weighed against the increased monetary, manpower, and animal welfare costs associated with amplified sampling of a very comprehensive study of respiratory physiology and molecular mechanisms. Specifically, samples from more than 140 individuals per treatment per time point (versus 8–16 individuals tested in our study) would be necessary to fulfill the requirements proposed by Mordecai et al. to achieve a statistical power of > 80% at the post hoc test. Overlooking this practical impossibility, we would argue that biological relevance of defining subtle virus manipulations afforded by improved post hoc comparisons would be minimal given the overarching allostatic ability for fish to maintain homeostatic functions as demonstrated in this study. For example, increasing confidence in asserting a transient ~ 8% reduction in mean Hct after a PRV injection does not make the observed change any more functionally relevant given our comprehensive biological knowledge of Hct in fishes [2, 6].

Likewise, we agree with Mordecai et al. that a set of unknown conditions could theoretically occur in natural environments for which PRV could potentially exploit to harmful effect on its host. Indeed, it could be argued that any ecological, biological, or chemical change imposed upon an organism creates a potential for harm. Where we disagree is that our current study suggests this risk would be more than minimal. Indeed, we would have had difficulty choosing a salmon virus with a more supportive literature for low virulence than the PRV isolate we used. Specifically, our PRV isolate (i) has not been known to cause disease in sockeye salmon even under circumstances of persistent high-load systemic infection, host saltwater adaptation, or viral co-infection [3, 8], (ii) has failed to cause disease in at least 4 other related salmon species in multiple saltwater and freshwater challenge trials conducted in the region [9–12], (iii) has shown minimal association (< 0.3%) and lack of direct causation for morbidity or mortality in commercial Atlantic salmon net-pen production in its endemic region [11, 13], (iv) is considered of low virulence to Norwegian Atlantic salmon and less impactful relative to endemic PRV in that region [14], and (v) has failed to induce respiratory consequences in Atlantic salmon [10] similar to what we identified in Sockeye. In consideration of cumulative evidence and literature, a formal risk assessment commissioned by the government of Canada has further concluded the risks posed to wild sockeye salmon from regionally endemic PRV used in our study to be less than minimal [15]. Our results identifying metabolic and functional maintenance during PRV infection are consistent with these earlier findings and lend support to this conclusion.

## Data Availability

All data supporting the conclusions of this article are presented within our original published article [2] or within Mordecai et al.’s correspondence [1] relating to that work.
